# Geldanamycin-Induced *Osteosarcoma* Cell Death Is Associated with Hyperacetylation and Loss of Mitochondrial Pool of Heat Shock Protein 60 (Hsp60)

**DOI:** 10.1371/journal.pone.0071135

**Published:** 2013-08-28

**Authors:** Magdalena Gorska, Antonella Marino Gammazza, Michal Aleksander Zmijewski, Claudia Campanella, Francesco Cappello, Tomasz Wasiewicz, Alicja Kuban-Jankowska, Agnieszka Daca, Alicja Sielicka, Urszula Popowska, Narcyz Knap, Jakub Antoniewicz, Takashi Wakabayashi, Michal Wozniak

**Affiliations:** 1 Department of Medical Chemistry, Medical University of Gdansk, Gdansk, Poland; 2 Department of Experimental Biomedicine and Clinical Neurosciences, Section of Human Anatomy “Emerico Luna”, University of Palermo, Palermo, Italy; 3 Euro-Mediterranean Institute of Science and Technology, Palermo, Italy; 4 Department of Histology, Medical University of Gdansk, Gdansk, Poland; 5 Department of Pathophysiology, Medical University of Gdansk, Gdansk, Poland; 6 Department of Pathology and Experimental Rheumatology, Medical University of Gdansk, Gdansk, Poland; 7 Department of Biochemistry, Medical University of Gdansk, Gdansk, Poland; 8 Department of Bioenergetics and Physiology of Exercise, Medical University of Gdansk, Gdansk, Poland; 9 College of Health, Beauty Care and Education in Poznan, Faculty in Gdynia, Gdynia, Poland; 10 Department of Cell Biology and Molecular Pathology, Nagoya University School of Medicine, Nagoya, Japan; University of Tennessee, United States of America

## Abstract

*Osteosarcoma* is one of the most malignant tumors of childhood and adolescence that is often resistant to standard chemo- and radio-therapy. Geldanamycin and geldanamycin analogs have been recently studied as potential anticancer agents for *osteosarcoma* treatment. Here, for the first time, we have presented novel anticancer mechanisms of geldanamycin biological activity. Moreover, we demonstrated an association between the effects of geldanamycin on the major heat shock proteins (HSPs) and the overall survival of highly metastatic human *osteosarcoma* 143B cells. We demonstrated that the treatment of 143B cells with geldanamycin caused a subsequent upregulation of cytoplasmic Hsp90 and Hsp70 whose activity is at least partly responsible for cancer development and drug resistance. On the other hand, geldanamycin induced upregulation of Hsp60 gene expression, and a simultaneous loss of hyperacetylated Hsp60 mitochondrial protein pool resulting in decreased viability and augmented cancer cell death. Hyperacetylation of Hsp60 seems to be associated with anticancer activity of geldanamycin. In light of the fact that mitochondrial dysfunction plays a critical role in the apoptotic signaling pathway, the presented data may support a hypothesis that Hsp60 can be another functional part of mitochondria-related acetylome being a potential target for developing novel anticancer strategies.

## Introduction

*Osteosarcoma* (OS) is one of the most aggressive malignant bone neoplasms of childhood and adolescence. Remarkable progress has been made in terms of improving surgical techniques combined with neoadjuvant and adjuvant chemotherapy, however, the 5-year survival rate plateaued at 50–70% [1], [2]. The main clinical problem with OS is high resistance to chemotherapy [3], [4]. Molecular chaperones of heat shock protein family (HSPs) play important roles in protein folding, translocation, as well as in cell proliferation, apoptotic signaling, and metastasis [5]–[7]. HSPs overexpression in the cell correlates with an increased risk of cancer development or resistance to chemotherapy [5]. Thus, HSPs are proposed to be considered as case-dependent specific tumor biomarkers [5], [8]–[11]. Moreover, they play a pivotal role in the cellular signaling, trafficking, and protein turnover. There are three major families of HSPs whose expression and activity are enhanced in cancer cells, namely the Hsp60, Hsp70, and Hsp90 families [12]. It seems that different cellular compartments such as mitochondria, cytoplasm or endoplasmic reticulum (ER) have a unique set of molecular chaperones. The most abundant eukaryotic HSP is Hsp90 as identified in two cytoplasmic isoforms: Hsp90AA1 (Hsp90 alpha, Hsp90a, HSPC1), Hsp90AB1 (Hsp90 beta, Hsp90b, HSPC3), and the ER homolog: Glucose-regulated Protein 94 (GRP94, Hsp90B1), all of them being expressed by distinct genes [13], [14]. In humans, there are several Hsp70 family members including stress-inducible Hsp70 (Hsp72, HspA1), and constitutively expressed HSC70 (Hsp73, HspA8) [15]. For many years Hsp60 was considered an exclusively mitochondrial chaperone. Surprisingly enough, Hsp60 was discovered in the cytoplasm, and extracellular compartment [16], [17].

Geldanamycin (GA) and geldanamycin analogs have been considered potential anticancer agents in OS treatment [18]. GA is a benzoquinone ansamycin antibiotic and a potent tyrosine kinase inhibitor that was first isolated from *Streptomyces hygrocopicus* in 1970. GA is also known as a potent inhibitor of Hsp90 [18]. GA binds specifically to the N-terminal ATP binding site of Hsp90 destabilizing the Hsp90 heterocomplexes with target proteins, and consequently leading to Hsp90-client proteins proteasomal degradation [18]. The mechanisms involved in GA-induced destabilization of Hsp90 complexes, and proteasomal degradation of Hsp90-client proteins are relatively well understood. However, very little is known about GA impact on modulation of HSPs expression, and potential correlation with chemotherapy effectiveness. It seems that GA influences HSPs expression by the stabilization of the transcription factor HSF1 trimeric complex with heat shock response element (HSE), and thus stimulates the expression of HSPs coding genes [19]–[21]. The therapeutic usage of GA has been limited mainly due to hepatotoxicity, however, less toxic GA analogs (17AAG, 17DMAG) have been developed, and extensively tested [22]–[24]. In this study, we aimed to investigate various effects of GA on OS 143B cell line focusing specifically on the viability, mode of GA-induced cell death, impact on HSPs gene expression, protein levels, and their distribution in distinct cellular compartments.

## Materials and Methods

### Reagents

Geldanamycin was purchased from InvivoGen (France). Tissue culture media, antibiotic cocktail, fetal bovine serum, dimethyl sulfoxide (DMSO) were purchased from Sigma-Aldrich (Poland). Horseradish peroxidase-conjugated antibodies against betabeta-actin, mouse monoclonal anti-Hsp90alpha/beta, mouse monoclonal anti-Hsp70 were purchased with Santa Cruz Biotechnology (Germany). Mouse monoclonal anti-Hsp60 antibodies were obtained from Sigma Aldrich (Italy). Rabbit polyclonal antibodies against Hsp90 alpha and rabbit monoclonal antibodies against Hsp90 beta were purchased from Abcam (UK). Rabbit polyclonal antibodies against acetylated lysine was obtained from Cell Signaling Technology (Italy). Goat anti-mouse and anti-rabbit IgG antibodies were purchased from Perkin Elmer (USA) and Abcam (UK), respectively.

### Cell line and culture conditions

The human OS 143B cell line was obtained from the American Tissue Type Collection (ATTC-8303). The cells were cultured at 37°C in a humidified atmosphere saturated with 5% CO_2_ using Dulbecco's Modified Eagle's medium supplemented with 10% heat-inactivated fetal bovine serum and a penicillin (100 µg/mL)/streptomycin (100 µg/mL) cocktail (Sigma-Aldrich, Poland).

### Cell treatment

Before each experiment, the cells were trypsinized with a solution of 0.25% trypsin and 0.02% EDTA, and were cultured for 24 h under the above-described conditions. The medium was then replaced with the one containing GA according to an experimental design. The stock solution of GA was prepared by dissolving the pure compound in DMSO. The final concentration of DMSO in the working solution was ∼0.001%. After an appropriate incubation time, the cells were harvested and analyzed by one of the methods described below.

### Cell viability assay (MTT assay)

OS 143B cells were seeded onto 96-well plates at a density of ten thousand cells/well and cultured for 24 h. Medium was then removed and cells were treated with serial GA dilutions (within the range of 0.8 µM–50 µM) of GA for 24 h. After the appropriate incubation time, 0.5 mg/mL of 3-[4,5-dimethylthiazol-2-yl]-2,5 diphenyltetrazolium bromide (MTT) was added. The plates were then incubated at 37°C for 4 h and the supernatant was removed. Finally, 100 µL of DMSO was added. Absorbance at 570 nm was determined using a microplate reader (Bio-Tek Instruments, Inc., USA). The number of cells was calculated based on absorbance values. The results were presented as a percentage of control. Each experiment was performed at least three times.

### Assessment of apoptosis by flow cytometry with double Annexin V - propidium iodide (PI) staining

OS 143B cells were seeded onto six-well plates at a density of three hundred thousand cells/well. After 24 h of culturing in the standard medium, the cells were treated with GA for 24 h. The cells were then pelleted and incubated with Annexin V and PI according to manufacturer's protocol (BD Pharmingen, Poland). Afterward, the cells (thirty thousand/sample) were analyzed, and the fluorescent signals of Annexin V conjugate and PI were detected at the fluorescence intensity channels FL1 and FL3 (BD FACScan). The results were then analyzed by Cyflogic software, version 1.2.1. Each experiment was performed at least three times.

### RNA extraction and Real Time PCR analysis

Reverse transcription (1 µg RNA/reaction) was performed with First Strand cDNA Synthesis Kit (Fermentas). RT PCR was performed using 5-fold diluted cDNA and RT PCR Master Mix (A&A Biotechnology). Reactions were performed with the initial denaturation at 95°C for 5 min (phase I) and then 40 cycles at 95°C for 15 sec, 57°C for 60 sec and 72°C for 20 sec, ending with fluorescence reading at 78°C for 10 sec (phase II). Melt Curve stage started with 95°C for 15 sec, next cooling to 70°C (redenaturation of present products) for 60 sec with temperature increasing to 95°C, fluorescence read each 0.3°C. The primers were designed based on the human OS DNA sequence with using Integrated Device Technology software. Real Time PCR reactions were performed employing Applied Biosystems StepOnePlus Real-Time PCR thermocycler and analyzed using StepOne Software. All qPCR reactions were repeated at least 3 times to ensure the replicability of the results.

The primers used were as follows:

Hsp90AA1 (F: AGGTTGAGACGTTCGCCTTTCA, R: AGATATCTGCACCAGCCTGCAA),

Hsp90AB1 (F: AGGAACGTACCCTGACTTTGGT, R: ATGCCAATGCCTGTGTCTACCA),

Hsp90B1 (F: TGGAGGAAGAAGAAGCAGCCAA, R: AAGTTCCCAGTCCCAGACAGTT),

Hsp70 (F: AAGGACATCAGCCAGAACAAGCGA, R: ACGTGTAGAAGTCGATGCCCTCAA),

Hsp60 (F: TTGCTACTGGTGGTGCAGTGTTTG, R: GCATGGCATCGTCTTTGGTCACAA).

### Western blotting

Equal amounts of total cell lysates were resolved by 9% SDS–PAGE and transferred to PVDF membranes (Amersham Biosciences) by means of semidry transfer (Bio-Rad). The membranes were then incubated with primary antibodies anti -total Hsp90, -Hsp70, -Hsp60, -Hsp90 alpha and -Hsp90 beta (1∶1000) overnight at 4°C. After having been washed 3 times per 5 min with TBS-T, the membranes were probed with horseradish peroxidase-conjugated goat anti-mouse or anti-rabbit IgGs (1∶50000) for 1 h at room temperature (RT). Subsequently, the membranes were washed 3 times with TBS-T. A visualization step was performed using a luminol-enhanced chemiluminescence (Chemiluminescence Blotting Substrate Kit, GE Healhcare, UK) according to the manufacturer's protocol. The protein level was quantified by densitometry technique using Quantity one 4.5.2 software. The protein levels, as determined by chemiluminescent quantification, were normalized relative to beta-actin levels found in the samples. Each experiment was performed at least three times.

### Immunoprecipitation

In order to detect protein complexes immunoprecipitation was performed as described by Campanella *et al.* [25]. Briefly, 5 µg of primary antibody (mouse monoclonal anti-HSP60, Clone LK1, Sigma-Aldrich) per 1 ml of total cell lysate, were incubated overnight at 4°C with gentle rotation. Antibody/protein complexes were then immunoprecipitated with antibodies linked to Sepharose A beads (Amersham Biosciences, Italy). Nonspecifically bound proteins were removed by repeated washing with isotonic lysis buffer. Immunoprecipitated proteins were resolved by 10% SDS-PAGE using acetylated lysine primary antibodies (Cell Signaling, Italy). Each experiment was performed at least three times.

### Immunofluorescent microscopy

143B cells were seeded onto an 8-well microscope chamber slide at the density of ten thousand cells/well, cultured for 24 h and then treated with GA. Cells were fixed with ice cold methanol for 30 min, washed in phosphate buffer solution (PBS); pH 7.4, and were incubated with the unmasking solution (trisodium citrate 10 mM, 0.05% Tween 20) for 10 min at RT. Then, the cells were incubated with the blocking solution (3% albumin bovine serum in PBS) for 30 min at RT and with primary antibodies (mouse monoclonal anti-HSP60, Clone LK1, Sigma-Aldrich; mouse monoclonal anti-HSP70, W27, Santa Cruz Biotechnology; mouse monoclonal anti-HSP90 alpha/beta, Santa Cruz Biotechnology, USA) diluted 1∶100, overnight at 4°C. The next day, the cells were incubated with the other primary antibody (rabbit polyclonal anti-AIF, H300, Santa Cruz Biotechnology) diluted 1∶100 overnight at 4°C for double immunofluorescence analysis.

After washing twice in PBS, the cells were incubated with fluorescent secondary antibodies such as mouse IgG antibody conjugated with FITC (Sigma-Aldrich, Italy), mouse IgG antibody conjugated with Texas Red (Sigma-Aldrich, Italy) both diluted 1∶200 for 1 h at RT. Moreover, rabbit IgG antibody conjugated with Texas Red (diluted 1∶200; Gene Tex Inc, Irvine, USA) was used for the detection of the immunofluorescent complexes. The nuclei were counterstained with Hoechst 33342 (Sigma-Aldrich, Inc, Italy) for 15 min at RT. Finally, all slides were mounted with cover slips using a drop of PBS, and then reading and imaging were immediately performed using a Leica DM5000 upright fluorescence microscope (Leica Microsystems, Heidelberg, Germany).

### Statistical analysis

Data from at least three independent experiments are presented as mean ± SE. Data were analyzed using GraphPad Prism (GraphPad Software, Inc., version 6.02, USA). Significant differences between groups were determined by One-way ANOVA combined with Dunett's Multiple Comparison test or Student's t-test. *P<0.01 was considered statistically significant.

## Results

### Effect of GA on proliferation of OS 143B cells

**Figure 1:**
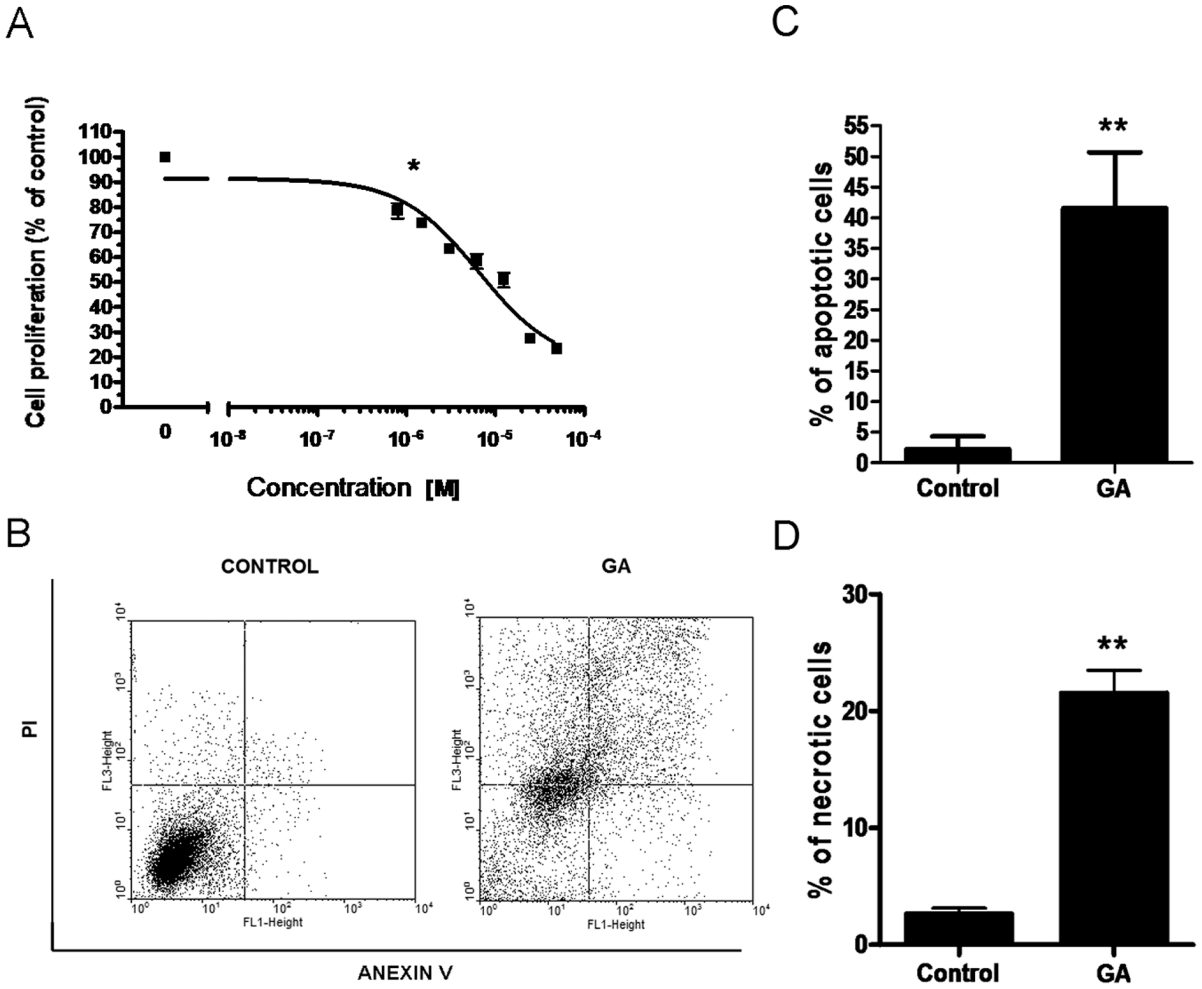
The effect of GA on proliferation and induction of cell death of OS 143B cells. **A**. GA inhibits OS 143B cell growth. OS 143B cells were treated for 24 h with serial GA dilutions (within the range of 0.8 µM–50 µM). The cell viability was then determined by means of MTT assay. Data from at least three independent experiments are presented as mean ± SE. The absence of error bar denotes a line thickness greater than the error. Data were analyzed by GraphPad Prism Software version 6.02 performing One-way ANOVA combined with Dunett's Multiple Comparison Test. *P<0.01 vs. control. **B–D**. GA induced cell death of OS 143B cells. 143B OS cells were treated with 4 µM GA for 24 h, the cells were then harvested and the percentage of apoptotic and necrotic cells was determined performing double PI-Annexin V staining. **B**. Annexin V, PI-live/dead dot plots showing apoptosis and necrosis before and after treatment with GA. Plots are representative of five individual experiments. **C–D**. Total apoptotic (C) and necrotic (D) cell number before and after treatment with GA. Data from at least three independent experiments are presented as mean ± SE. Data were analyzed using GraphPad Prism (GraphPad Software, Inc., version 6.02, USA). Significant differences between groups were determined by Student's t-test. *P<0.01, ***P<0.0001 vs. control.

One of the main goals of the study was to determine the inhibitory effect of GA on highly metastatic human OS 143B cell line. The cells were treated for 24 h with serial GA dilutions (within the range of 0.8 µM–50 µM), Subsequently, the inhibition of 143B cell growth was assessed by means of MTT-assay. GA effectively inhibited OS cell growth in a concentration-dependent manner. Viability of 143B cells was significantly decreased from 77% to 23% in the presence of growing concentrations of GA as compared to control (Figure 1A).

### GA induced apoptosis and necrosis in OS 143B cells

In order to determine the influence of 24 h treatment of 143B OS cells with GA on the induction of cell death, flow cytometric-double staining (Annexin V and PI) was performed. An increase in apoptotic and necrotic cells was observed after GA (4 µM) treatment (Figure 1B–D). The percentage of apoptotic 143B cells found after 24 h long treatment with GA (4 µM) was equal to 41.5%±6% (Figure 1C), while percent of necrotic cells was equal to 20.9%±4.5% (Figure 1D). Precisely, 23.9%±2.5% and 17.6%±3.5% were found in the early stage and, in the late stage of apoptosis, respectively (Figure 1B). Moreover, the treatment of 143B cells with 4 µM GA for 6 h resulted in the upregulation of apoptosis-inducing factor (AIF) as shown by immunofluorescence (see **Impact of GA on Hsp60 level and gene expression**).

### Impact of GA on Hsp90 gene expression and protein level

**Figure 2:**
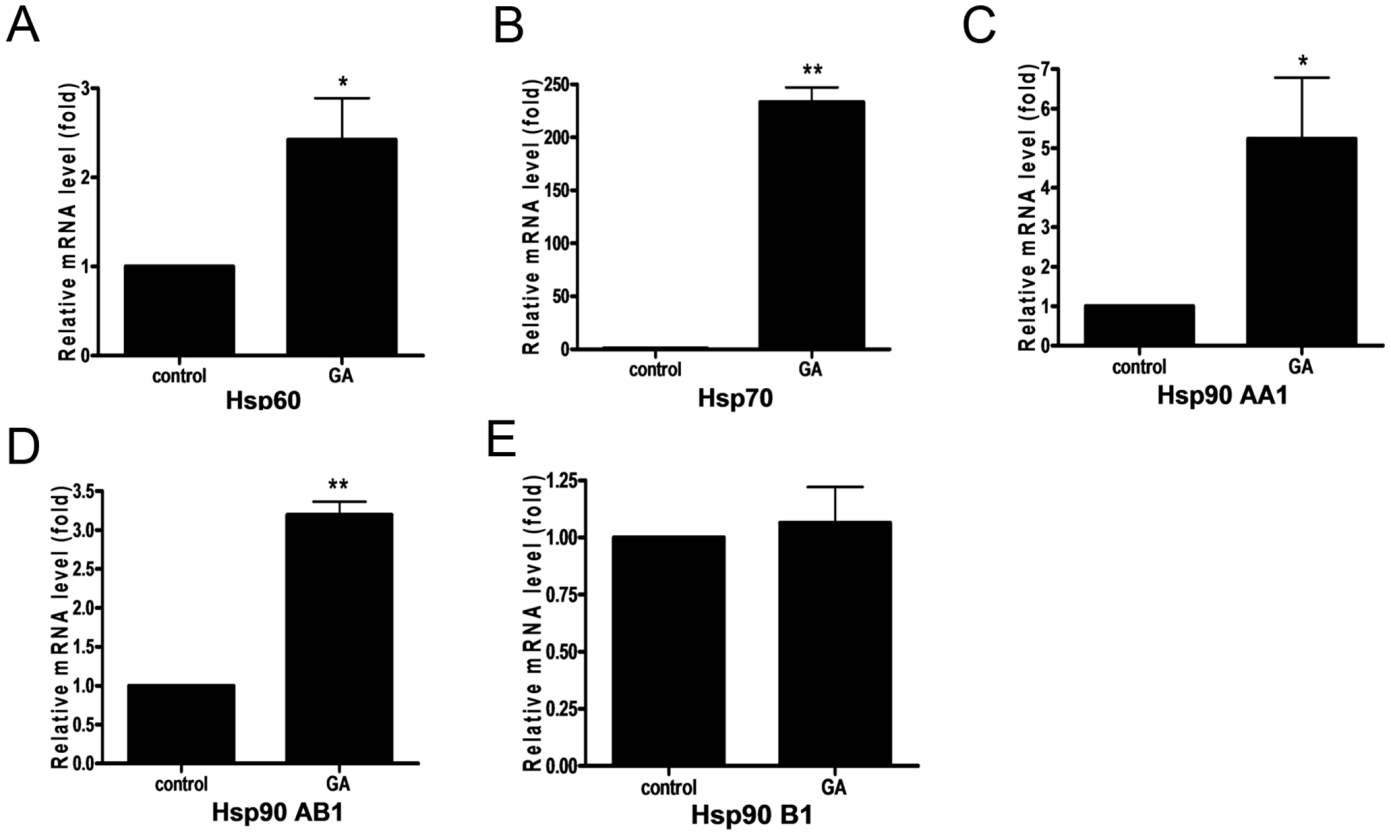
GA affected HSPs gene expression in OS 143B cells. OS 143B cells were treated with GA (4 µM) for 6 h; the levels of Hsp60, Hsp70, Hsp90AA1, Hsp90AB1, Hsp90B1 transcript were then determined by means of Real Time PCR. Treatment with GA resulted in upregulation of Hsp60 (A), Hsp70 (B), Hsp90AA1 (C), Hsp90AB1 (D) transcript, however it did not impact Hsp90B1 gene expression (E). Values are mean ± SE of three independent experiments, relative mRNA levels of HSP/beta-actin are presented. The data were analyzed by Student's t-test using GraphPad Prism Software version 6.02. *P<0.01, **P<0.001 vs. control.

**Figure 3:**
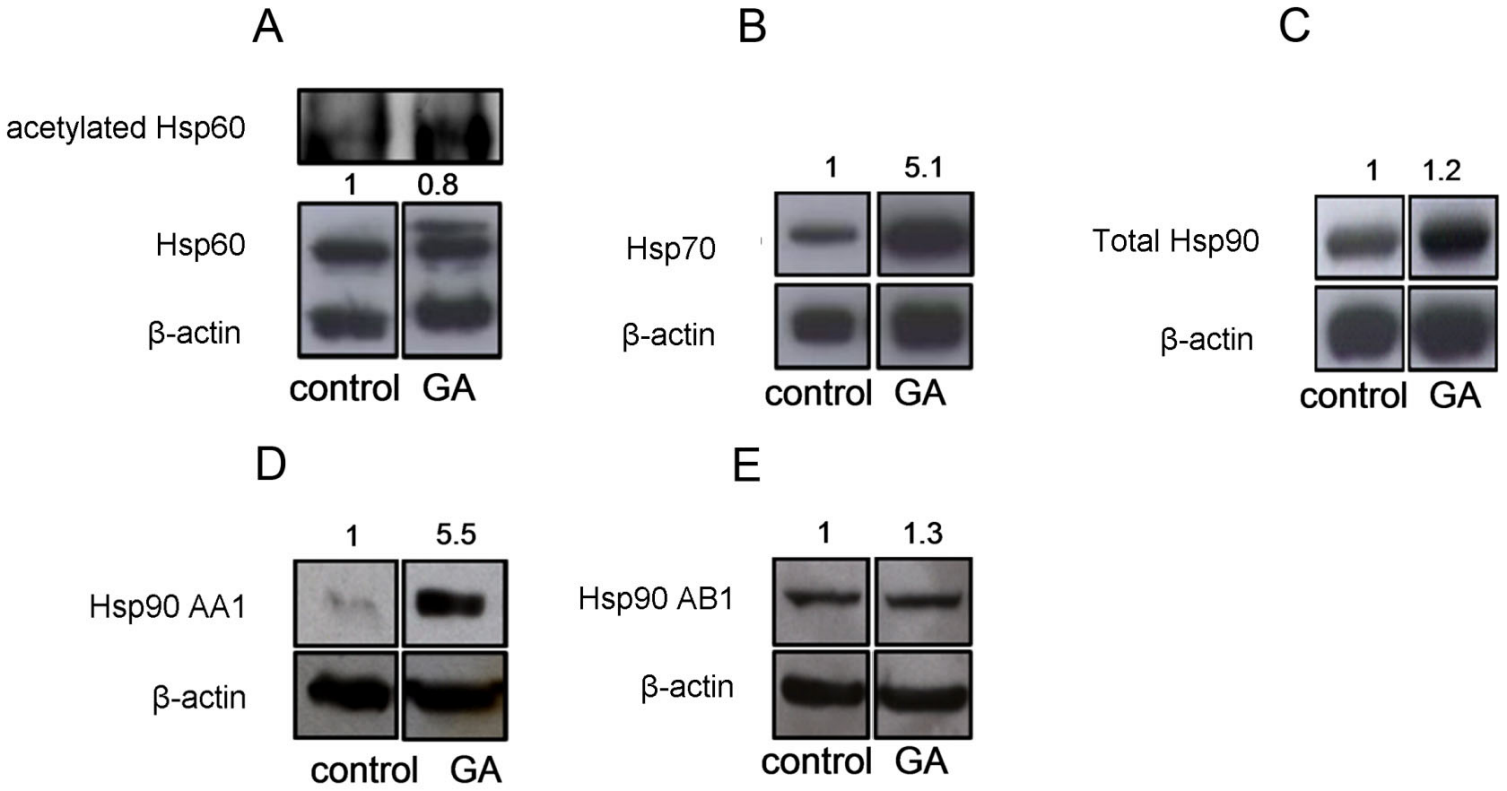
GA affected HSPs protein levels in OS 143B cells. OS 143B cells were treated with 4 µM GA for 6 h; the levels of Hsp60, Hsp70, total cellular Hsp90, Hsp90AA1, Hsp90AB1proteins were determined by Western blotting. **A**. GA decreased the level of Hsp60 and induced post-translational modification of Hsp60 partially derived from the hyperacetylated isoform. **B–E**. GA upregulated Hsp70 (B), total cellular Hsp90 (C), Hsp90AA1 (D), Hsp90AB1 (E) protein levels. Each experiment was performed at least three times. The representative data are shown. Densitometric analysis of HSP/beta-actin was performed using Quantity one 4.5.2 software.

**Figure 4:**
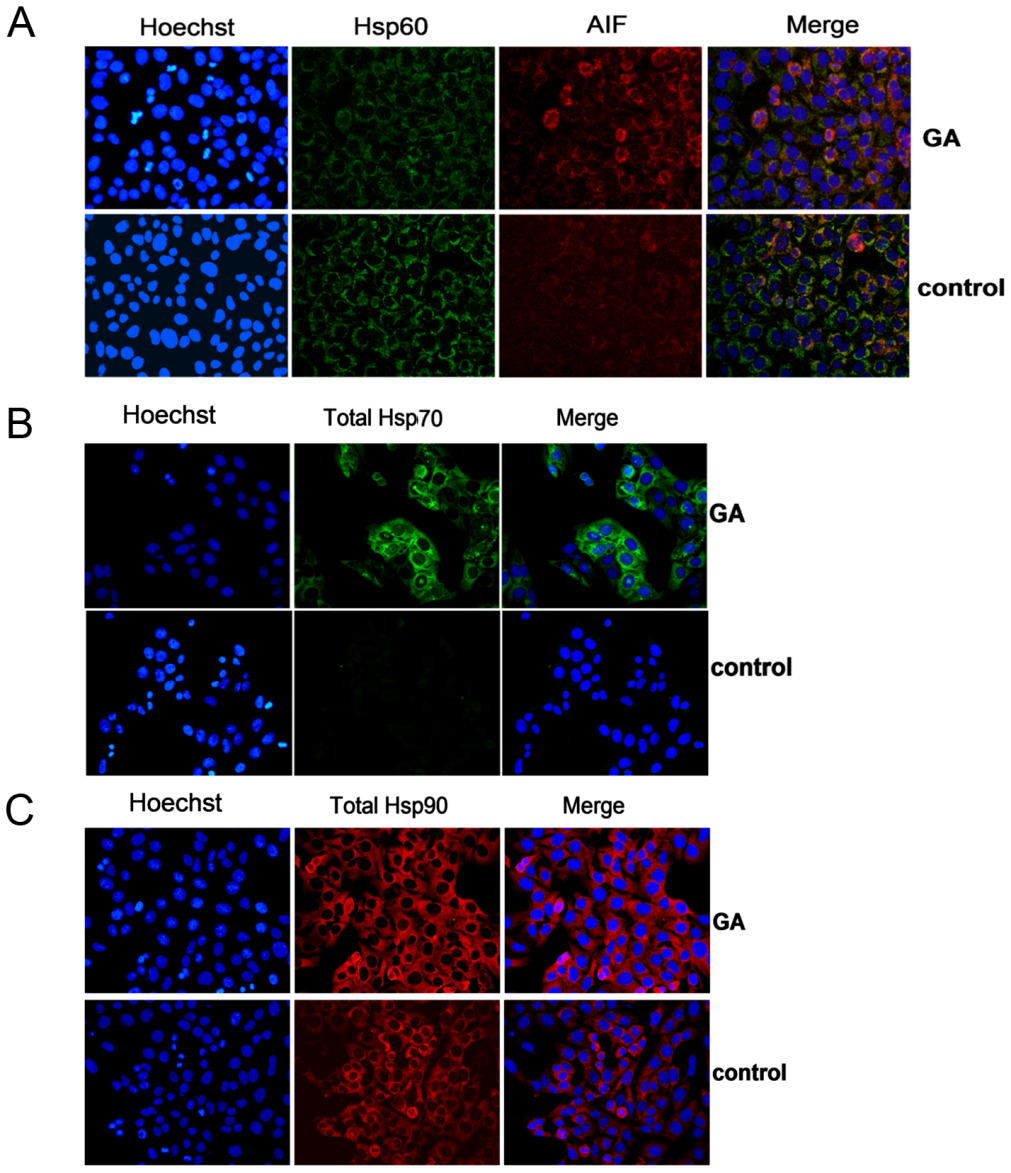
Effect of GA on localization and protein level of HSPs in OS 143B cells. OS 143B cells were treated with 4 µM GA for 6 h; the levels of Hsp60, Hsp70, total cellular Hsp90 protein were determined by immunofluorescence. **A**. GA did not change the mitochondrial localization of Hsp60, however, it decreased the mitochondrial pool of the protein. Cell nuclei were shown in blue while Hsp60, AIF immunoreactivities in green and red, respectively. Merged image of all kinds of staining (orange) was also presented. **B**. GA upregulated Hsp70 protein level. Cell nuclei were shown in blue, while Hsp70 immunoreactivity in green. Merged image of both was also presented. **C**. GA upregulated Hsp90 protein level. Cell nuclei were shown in blue while Hsp90 immunoreactivity in red. Merged image of both was also presented. Original magnification ×40. Each experiment was performed at least three times. The representative data were shown.

GA is known to bind specifically to the ATP-binding site of Hsp90AA1 and Hsp90AB1 with similar affinities which results in the inhibition of Hsp90s interaction with the target proteins [18]. In order to investigate whether GA affects Hsp90 gene expression, 143B cells were treated with GA (4 µM) for 6 h. Subsequently, Hsp90AA1, Hsp90AB1, and Hsp90B1 mRNA levels were analyzed by means of Real Time PCR. Interestingly, GA caused 3- and 5-fold increase in the level of HspAB1 and Hsp90AA1 transcript, respectively (Figure 2C–D). However, GA did not significantly affect ER Hsp90B1 isoform (Figure 2E). The levels of the studied Hsp90 proteins, as observed in 143B cells after treatment with GA (4 µM) for 6 h, were also determined by Western blotting. The obtained data were in accord with those from Real Time PCR. The treatment of 143B cells with GA (4 µM) for 6 h resulted in a 1.2-, 5.5-, and 1.3-fold increase in the protein levels of the total cellular Hsp90, Hsp90AA1, and Hsp90AB1, respectively, as compared to control (Figure 3C–E). The effect of GA (4 µM) on the total cellular Hsp90 expression was then confirmed by immunofluorescence. Five random fields were acquired for each experimental condition (the representative picture is presented in Figure 4C). Cell nuclei were stained by Hoechst 33342 and shown in blue, Hsp90 immunoreactivity was shown in red. Treatment of 143B cells with GA (4 µM) remarkably increased the total cellular Hsp90 as compared to control (see **Impact of GA on Hsp60 level and gene expression**).

### Impact of GA on Hsp70 level and gene expression

GA can activate HSF1 which results in the induction of Hsp70 as previously demonstrated in certain experimental models, e.g. K562 erythroleukemic or rat 9L gliosarcoma cells [19], [21]. GA-induced upregulation of Hsp70 has been recently linked to chemotherapy resistance [15], [26]–[28]. In this study, we demonstrated that OS treatment with GA (4 µM) for 6 h resulted in a 233-fold increase in the Hsp70 transcript level as compared to control (Figure 2B). These results were in accord with the elevated Hsp70 protein levels as shown by Western blotting (Figure 3B). A 5-fold increase in the protein level of Hsp70 was observed in response to 6 h treatment with GA (4 µM) (Figure 3B). The upregulation of Hsp70 protein level by GA (4 µM) in 143B cell line was also visualized using immunofluorescence technique. Five random fields were acquired for each experimental condition (the representative picture is presented in Figure 4B). Cell nuclei were stained by Hoechst 33342 and shown in blue, Hsp70 immunoreactivity was shown in green. The obtained results showed a significant increase in the Hsp70 protein level as observed in GA-treated cells (Figure 4B).

### Impact of GA on Hsp60 level and gene expression

Not much is known about the effects of GA on the mitochondrial chaperone Hsp60, involved in the regulation of OS cell proliferation [9]. Thus, our goal was to determine the impact of GA on Hsp60 gene expression. Treatment with GA (4 µM) resulted in a 2.4-fold induction of Hsp60 gene expression in 143B cells after 6 h incubation (Figure 2A). On the other hand, the data obtained from immunofluorescense analysis indicated decreased Hsp60 protein level observed after 6 h treatment of 143B cells with GA as compared to control (Figure 3A). The effect of GA was then confirmed by Western blotting. Interestingly, a 20% decrease in the level of Hsp60 in GA-treated 143B cells (Figure 3A). Moreover, after 6 h treatment with GA, an extra band corresponding with slightly higher molecular weight was identified by standard Western blotting. The occurrence of some additional immunoreactivity suggested a possible post-translational modification of Hsp60 after treatment with GA (Figure 3A). Interestingly, Singh *et al.* [29] demonstrated that cytosolic and mitochondrial forms of Hsp60 differed in their molecular weight. That is why, the exact localization of Hsp60 within the cellular compartments of OS 143B was investigated before and after GA treatment. Mitochondrial localization of Hsp60 was confirmed using double-labeled immunofluorescent microscopy. AIF played a role of natural marker of mitochondria. Five random fields were acquired for each experimental condition (the representative picture is presented in Figure 4A). Cell nuclei were stained by Hoechst 33342 and shown in blue, Hsp60 immunoreactivity was shown in green, AIF immunoreactivity in red. The merge of Hsp60, AIF and cell nuclei (orange) was also presented. It was demonstrated that GA (4 µM) did not influence Hsp60 localization, which suggests that the extra band visualized by Western blotting is not the cytosolic Hsp60 (Figure 4A). One of the essential post-translational modification affecting HSPs function is their hyperacetylation [31]. In order to determine if hyperacetylation was induced by GA, a double-labeled immunoprecipitation of acetylated lysine and Hsp60 was performed. Interestingly, the obtained results confirmed that the additional band of Hsp60 as visualized on Western blot might at least to some extent derive from GA-induced post-translational hyperacetylation of Hsp60 protein.

## Discussion

Previous reports have clearly established that Hsp90 and other chaperones are required for survival of both normal and malignant cells, and have served as useful cancer biomarkers [5], [31]. OS is one of many cancers with poor prognosis, and the resistance of OS to chemotherapy is partly associated with overexpression of HSPs [11]. The upregulation of HSPs is believed to be tied in to tumor progression, sustained cancer cell proliferation, development of drug resistance and long-term cellular adaptation [32], [33]. In our study, we followed the effects of GA on the major HSPs (Hsp90, Hsp70, Hsp60) localized in distinct cellular compartments (cytosol, mitochondria, ER) of highly metastatic OS 143B cells. Our findings demonstrated that GA is a potent inhibitor of 143B cell growth and programmed cell death inducer (Figure 1A–D) suggesting that it could be potentially effective in OS chemotherapy. It has been known that GA inhibits the activity of Hsp90. However, the molecular mechanism leading to GA-induced cell death still remains to be elucidated. Kim *et al.* [19] presented that GA activated HSF1 transcription factor, which resulted in the upregulation of HSPs gene expression. The modulation of HSPs expression was later confirmed by Sittler *et al.* [34] showing the upregulation of Hsp70 and Hsp90 in COS-1 cells treated with GA. Here, we presented that the anticancer activity of GA seemed to be associated with GA impact on the mitochondrial and cytosolic expression of the major HSPs. According to our data GA upregulated cytosolic isoforms of Hsp90 (Hsp90AA1, Hsp90AB1) whereas it had no effect on ER Hsp90B1 level in GA-treated 143B cells (Figure 2C–E). GA treatment resulted in a comparable induction of Hsp90AA1 gene expression and protein synthesis in 143B cells (Figure 3D), whereas GA-stimulated transcription of Hsp90AB1 gene did not correlate with Hsp90AB1 protein level as observed in 143B cells (Figure 3E). This finding might suggest different control mechanisms of Hsp90AB1 protein synthesis that do not correlate with mRNA levels. It is not surprising in light of higher inducible potential of Hsp90AA1 as observed in the rat 9L gliosarcoma cells [13]. Nevertheless, the impact of GA on 143B cells seems to be dependent on the cell line and GA concentration. Chang *et al*. showed diminished expression of Hsp90AA1 in comparison with Hsp90AB1 after 5 h treatment with 0.5 µM GA which contradicts *de novo* synthesis of the studied proteins as observed in rat gliosarcoma 9L cells [13]. In the urinary bladder cancer cells, Karkoulis *et al.* showed the downregulation of the total cellular Hsp90 caused by 17-AAG at concentrations below 0.1 µM, while the upregulation was observed at concentrations above 1 µM [35]. Furthermore, we presented that GA treatment highly induced Hsp70 gene expression. (Figure 2, 3, 4B). Regarding recent findings in terms of pharmacological induction of Hsp70 and Hsp90 as one of the principal factors leading to cancer development, metastasis and drug resistance [36], [8], as well as the HSPs upregulation observed in our studies, we decided that it might be of importance to determine the impact of GA on yet another essential HSPs protein, namely Hsp60. It was proven that the Hsp60 knockdown contributed to OS cell arrest [9], [37]. For the first time we demonstrated that GA treatment of OS 143B cells resulted in cell death (Figures 1A–C), which seems to coincide with hyperacetylation and decreased level of mitochondrial Hsp60 (Figure 3–4A). Hsp60 (as well as many other chaperones) is known to form numerous complexes, and the deregulation of Hsp60 activity, possibly via acetylation, can disturb protein folding in the cell [37], and cause proteotoxic stress ultimately resulting in the switch of prosurvival signaling to cancer cell autoelimination [38]. Our previous data strongly suggest that acetylation of interphase microtubules in OS 143B cells can be an essential molecular mechanism of mitochondrial biogenesis regulation [38]. Interestingly, reversible hyperacetylation was shown to deregulate the Hsp90 and Hsp70 chaperone functions, and to affect a great number of mitochondrial metabolic pathways [5], [30]. There are no data concerning the regulation of Hsp60 activity via acetylation, however, Sol et al. demonstrated that Hsp60 was one of the proteins undergoing acetylation in U2OS OS cells with sirtuine 3 (Sirt3) knockout [39]. Sirtuins play an essential role in the regulation of mitochondrial oxidative stress and tumorigenesis [39]–[41]. In 2007, Lombard *et al.* suggested that Sirt3 knockdown led to substantial hyperacetylation of the mitochondrial protein [41]. On the other hand, protein deacetylation has been found to enhance xenobiotic liver injury by modulating the binding of toxic metabolite of acetaminophen to the mitochondrial protein [42]. Such a strategy can be applied in the treatment of tumors using hepatotoxins like acetaminophen as anticancer enhancers [43]. That is one of the major reasons why sirtuins and other deacetylases have been recently investigated in order to elucidate their potential role in GA-induced hyperacetylation of Hsp60.

The process of hyperacetylation of Hsp60 as well as other chaperones (Hsp70 and Hsp90) seems to be an emerging target for chemotherapeutic treatment of patients presenting with OS. GA and GA less toxic derivatives might be good candidates for combined adjuvant and/or neoadjuvant chemotherapy.
